# Primary Liver Rhabdomyosarcoma with Burnt-out Paravertebral Disease

**DOI:** 10.1007/s12098-024-05399-1

**Published:** 2025-01-11

**Authors:** Vadisetti Satya Niharika, Shraddha Patkar, Trupti Pai, Sajid Qureshi

**Affiliations:** 1 Department of Surgical Oncology, Tata Memorial Hospital, Homi Bhabha National Institute, Mumbai, 400012 India; 2 Department of Pathology, Tata Memorial Hospital, Homi Bhabha National Institute, Mumbai, India

**Keywords:** Liver, Rhabdomyosarcoma, Pediatric

## Abstract

Less than 20 cases of primary hepatic rhabdomyosarcoma have been reported in literature. The authors present this occurrence in a neonate with paravertebral and peritoneal disease. Histopathology was the solution to authors’ diagnostic dilemma. Multimodality management is the solution for rare cases where specific guidelines are not yet defined. This is a rare case reporting a primary liver rhabdomyosarcoma with peritoneal disease with excellent survival.

## Introduction

Rhabdomyosarcoma (RMS) is the most common soft tissue sarcoma in pediatric age group, however primary intrahepatic RMS is very rare. Since 1956, less than 20 cases of hepatic RMS have been reported across all age groups [1, 2].

## Case Report

The child first presented to authors as a neonate at 15 d with a left lumbar mass. She was the second born from a non-consanguineous marriage with a healthy elder sibling, belonging to low socio-economic status with no significant family history. Contrast enhanced computed tomography of abdomen showed a large heterogenous mass in the left paravertebral location extending from D8 – L5 vertebral level with invasion of the psoas and paraspinal muscles. A single hypodense lesion was seen in segment IV of liver measuring 3.2 × 2.5 cm. A diagnosis of infantile neuroblastoma was made but she was lost to follow-up during Covid-19 pandemic.

The child was brought for further management at age of 16 mo with fever, hepatomegaly and severe malnutrition. Tumor markers showed elevated LDH – 596 IU/ml and ferritin – 67.03 mg/dl.

**Fig. 1:**
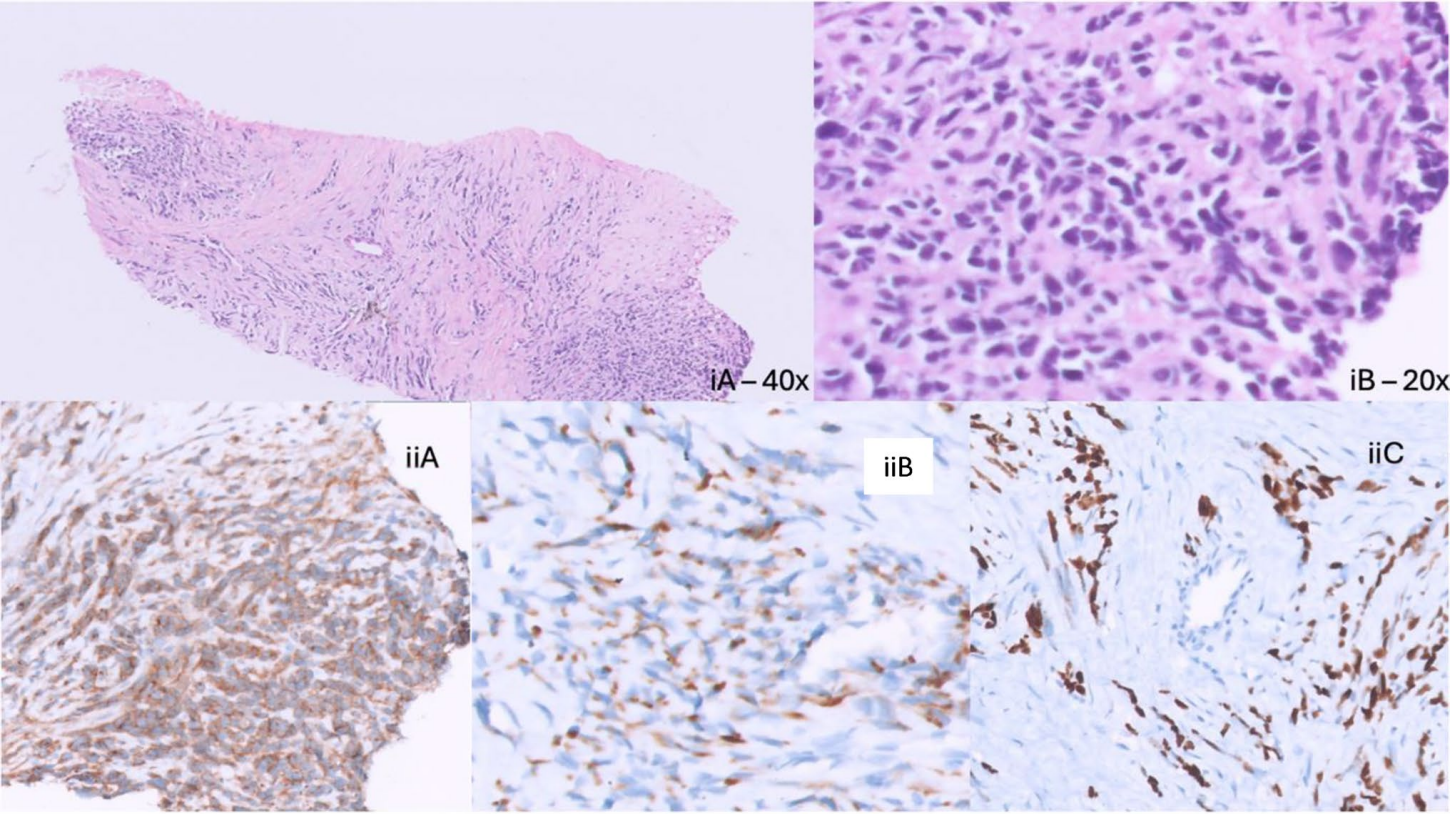
Photomicrograph of hematoxylin and eosin images of liver mass showing a spindle tumour, **(iA)** and **(iB)** IHC images of the liver mass biopsy: **(iiA)** Diffuse, dot-like and incomplete membranous reactivity for MIC2, **(iiB)** Desmin positive, **(iiC)** Diffuse, strong positivity for MyoD1

Positron emission tomography scan showed a 9.4 × 9 × 11 cm 18-fluorodeoxyglucose avid heterogeneously enhancing mass with central necrosis and peripheral enhancement involving all segments of liver except segment 6, para-aortic nodal mass, sub-hepatic peritoneal deposits and a splenic lesion. The residual paravertebral soft tissue mass was seen to left of D10 – L2 vertebra that was non-avid on meta-iodobenzylguanidine nuclear scintigraphy scan. Bone marrow aspiration and biopsy were negative for disease. Image guided biopsy of the paravertebral mass showed retroperitoneal fibrosis and the liver mass was reported as RMS. Immunohistochemistry panel showed positivity for desmin, myogenic determination factor 1 (MyoD1), incomplete membranous and dot like positivity of Synonym of CD 99 (MIC2) while negative for synaptophysin and S100. Molecular analysis was negative for PAX3-FKHR and PAX7-FKHR gene translocation (Fig. 1). Paravertebral disease was decided to be spontaneous regression of possible benign etiology based on the clinico-investigational picture in a multi-disciplinary team meeting.

The child received six cycles neoadjuvant VCD regimen (Vincristine, Cyclophosphamide and Actinomycin D). A computed tomography scan for response assessment showed only a 4.7 × 3.8 × 3.6 cm segment 4 hypodense liver lesion with stable paravertebral lesion. She underwent a left hepatectomy and omental nodule reported negative for malignancy on frozen section. The gross specimen revealed a whitish fibrotic liver lesion. Extensive therapy related changes were noted in the form of fibrosis, hyalinization and histiocytic infiltration, without any residual tumor. In view of previous paravertebral and peritoneal disease, whole abdominal radiotherapy with a dose of 25.2 GY in 14 fractions using intensity modulated radiation therapy was given followed by 28 wk adjuvant VCD regimen. The child is now disease free at 48 mo from surgery.

## Discussion

Primary RMS of the liver is a rare and commonly misdiagnosed entity due to its non-specific presentation and uncommon location. Lack of characteristic clinical parameters or imaging makes histopathology the gold standard for diagnosis [2]. Though no single specific tumor marker has been identified, lactate dehydrogenase (LDH) is widely used as it is reported to be a negative prognostic marker [3, 4]. 

The previous case reports showed a mean survival time of 9.25 mo (2–9) with a single report showing survival of 38 mo. This is an unique case reporting primary liver rhabdomyosarcoma with peritoneal disease with excellent survival. Of all hepatic RMS cases reported, the mean survival time of patients who underwent surgery is significantly better than those who were treated with non-surgical modalities. Most of the surgeries were anatomical resections (left/right hepatectomy or extended hepatectomy) based on the size and the site of the tumor, and there was no significant difference in surgical methods.

Tumor histology affects survival with embryonal subtypes having better prognosis than pleomorphic, spindle cell and alveolar varieties [5]. Further sub-types which differ in behavior and morphology were introduced based on molecular markers [6]. Malempati and Hawkin suggested risk stratification of RMS based on tumor size, invasiveness, nodal status, primary tumor site, pathological and PAX-fusion status [7].

Multi-modality management includes surgery, radiation therapy, and chemotherapy. Neoadjuvant chemotherapy not only helped to curtail metastatic spread but also to reduce the tumor size that improved future liver remnant and allowed complete tumor resection. Based on authors’ experience and previous case reports, multimodality management might be a key factor affecting survival.
